# Assessment of gene signatures following the inhibition of IL-23: a study to evaluate the mechanistic effects behind the clinical efficacy of guselkumab in patients with psoriatic arthritis

**DOI:** 10.3389/fimmu.2025.1672667

**Published:** 2025-09-02

**Authors:** Mirco Mastrangelo, Piero Ruscitti, Manfredo Bruni, Eleonora Lucantonio, Andrea De Berardinis, Antonio Barile, Maria Concetta Fargnoli, Paola Cipriani, Maria Esposito, Cristina Pellegrini

**Affiliations:** ^1^ Department of Biotechnological and Applied Clinical Sciences, University of L’Aquila, L’Aquila, Italy; ^2^ San Gallicano Dermatological Institute, Istituto di ricovero e cura a carattere scientifico (IRCCS), Rome, Italy

**Keywords:** psoriasis, psoriatic arthritis, guselkumab, therapy, transcriptome

## Abstract

**Objectives:**

The aim of this study was to evaluate the transcriptome of peripheral blood mononuclear cells (PBMCs) derived from patients affected by psoriasis (PSO) and psoriatic arthritis (PSA) following treatment with guselkumab, an interleukin (IL)-23 inhibitor.

**Methods:**

mRNA was extracted by PBMCs, before and after 24 weeks of treatment with guselkumab, and RNA sequencing was performed in paired-end mode by Illumina technology using the Novaseq6000 platform. log2FoldChange > 1 and *p*_adj_ < 0.05 were the established cutoff to discriminate genes differentially expressed between pre- and post-therapy. For annotation and predictive enrichment analysis of deregulated genes in biological pathways, RStudio was used, and Gene Ontology (GO) and Kyoto Encyclopedia of Genes and Genomes (KEGG) databases were queried. Cytoscape_v3.10.3 was used for the development of the network between deregulated genes.

**Results:**

Six naïve active patients with PSO and PSA, diagnosed with a duration <2 years, were assessed before and after 24 weeks of treatment with guselkumab. Performing the quality check and filtering analyses, we found 506 transcripts deregulated between pre- and post-therapy, of which 129 were upregulated and 377 were downregulated. The most upregulated mRNAs included SYTL3, CPT1A, TMEM208, GINS4, and TNFRSF13C. The most downregulated mRNAs included CCR2, TPT1, MYCBP, CMPK1, and TMEM65. Enrichment with the GO database showed the following main deregulated processes: “protein targeting”, “the establishment of protein localization to membrane”, and “metabolism of fatty acids”. The analysis of the main macro-process showed the several pathways deregulated after therapy, including signaling related to ethanol metabolism, thermogenesis, oxidative phosphorylation, and fatty acid metabolism (KEGG).

**Conclusions:**

Our findings may give further insights into manipulated mechanistic pathways by IL-23 inhibition in patients with PSO and PSA.

## Introduction

Psoriasis (PSO) is an inflammatory skin disease characterized by well-defined erythematous and scaly plaques mainly localized on the extremities and the scalp, which can be associated with musculoskeletal manifestations, involving peripheral joints, entheses, and axial skeleton, under the umbrella of the so-called psoriatic arthritis (PSA) (1, 2). In this context, the idea of the psoriatic disease continuum has been progressively prompted based on the advances of the knowledge about the occurrence of PSO and PSA (3). A complex and multidimensional pathogenetic model has been recently proposed linking the inflammatory mechanisms underlying both skin and joint manifestations (4, 5). On a predisposing genetic background, a combination of environmental triggering factors, such as smoking, biomechanical stress, infections, or obesity, and an aberrant immune response may induce the development of a chronic inflammatory process and aberrant production of inflammatory cytokines (6). The resulting dysregulation of the innate and adaptive immune system, underpinning the disease development, may simultaneously involve both the skin and the joints, leading to the clinical manifestations (7–9). In this context, multiple lines of evidence show the importance of interleukin (IL)-23 in the pathogenesis of psoriatic disease since it may induce and perpetuate the activity of Th17 cells (4–9). This heterodimeric cytokine is composed of IL - 12B (IL - 12p40) and IL - 23A (IL - 23p19) subunits. Given this pathogenic background, IL - 23 inhibitors have been successfully used to treat patients with PSO and PSA (10, 11). The administration of these drugs is suggested in patients, who are identified as non-responders to first-line therapies (12, 13). Among IL - 23 inhibitors, guselkumab specifically targets the p19 subunit of IL - 23; it shows a high rate of efficacy in patients with PSO and in those with PSA (10–13). However, the mechanisms underlying the efficacy of guselkumab are not fully elucidated yet. In this context, bulky RNA sequencing of peripheral blood mononuclear cells (PBMCs) could provide the landscape of mechanisms associated with the efficacy of guselkumab in these patients (14, 15) and how the inhibition of IL - 23 could also affect other pathways in psoriatic disease, resulting in patient clinical response. On these bases, we aimed at evaluating the data of RNA sequencing of PBMCs derived from patients affected by PSO and PSA following treatment with guselkumab.

## Methods

### Study design and patients

Consecutive active patients diagnosed with PSO and PSA (16, 17) were assessed among those attending the Dermatologic and/or Rheumatologic Clinics of the University of L’Aquila, L’Aquila, Italy (16, 17). We included adult patients (age ≥18 years) with PSO, naïve to systemic immunomodulating therapies, diagnosed to have PSA with a duration <2 years, and eligible to treatment with an IL - 23 inhibitor since they were affected by a moderate to active disease evaluated by the Psoriasis Area Severity Index (PASI) for skin involvement and Disease Activity Index for Psoriatic Arthritis (DAPSA) for joint manifestations. We included patients with PSO and PSA to find possible common pathogenic mechanisms, across the concept of the psoriatic disease continuum (1–5), in a hypothesis generating exploratory work to provide the basis for further studies. Considering the route of administration, following the induction phase, guselkumab was given every 8 weeks. In these patients, blood samples were collected before and after 24 weeks of treatment with guselkumab. PBMCs were obtained from 10 mL of whole blood by standard protocols and stored at −80°C.

The Internal Review Board of the University of L’Aquila (protocol number: Internal Review Board University of L’Aquila 01/2022) approved this study.

### RNA extraction

Total RNA was extracted at baseline and after treatment with guselkumab from PBMCs, by using the AllPrep DNA/RNA/miRNA Universal kit (Qiagen, Germany) according to the manufacturer’s instructions. RNA quantification and purity were measured with Qubit Fluorometer v3.0 (Thermo Fisher Scientific). One microgram of RNA was subjected to retro-transcription by using the TaqMan^®^ Micro-RNA Reverse Transcription kit (Thermo Fisher Scientific, CA, USA).

### RNA sequencing

RNA sequencing was performed in paired-end mode by Illumina technology using the Novaseq6000 platform. A quality check was performed on the sequencing data using the FastQC tool (18). The reference track was the assembly Human obtained from GenCode (relese35-v44) (19, 20) for human samples.

The quantification of transcripts expressed for each sequenced sample was performed using the featureCount algorithm. The DESeq2 tool (21) was used to normalize the data and perform the differential expression analysis. In this case, we performed PostTherapy (PostT) vs. PreTherapy (PreT) comparison. log2FoldChange > 1 and *p*_adj_ < 0.05 were used to discriminate genes that were differentially expressed. For annotation and predictive enrichment analysis of deregulated genes in biological pathways, Gene Ontology (GO) term ontologies and Kyoto Encyclopedia of Genes and Genomes (KEGG) 2021 Human were queried trough RStudio (22). Cytoscape_v3.10.3 and ClueGO were used for the development of the network between deregulated genes.

## Results

### Clinical findings

Six patients (mean age, 54.5 ± 9.9 years; four women and two men) with PSO and PSA, diagnosed for a duration <2 years, were assessed before and after 24 weeks of treatment with guselkumab. All these patients were naïve to systemic immunomodulating therapies and eligible to treatment with an IL - 23 inhibitor according to clinician judgment since all were affected by a moderate to active disease considering both skin and joint involvement. All patients were affected by moderate to severe plaque-type PSO and underwent current topical therapies (mean PASI, 3; range, 1.5–15). All patients were also affected by peripheral arthritis (mono- or oligoarthritis), enthesitis, and axial involvement. Before the administration of guselkumab, they were treated with nonsteroidal anti-inflammatory drugs (NSAIDs) but without a good clinical response (mean DAPSA, 18; range, 14–26). All patients were treated with guselkumab in monotherapy. Following the administration of such drug for 24 weeks, all patients achieved a clinically relevant response for the skin involvement with an almost complete remission of the lesions (achievement of PASI90). Considering the joint manifestations, all patients but one attained a clinically significant response during the follow-up (DAPSA < 4). The non-responder patient did not present differences compared to the other patients and did not reach the clinical target for the joint involvement (DAPSA > 14). No side effects were reported in these patients.

### Bulky RNA sequencing

**Figure 1:**
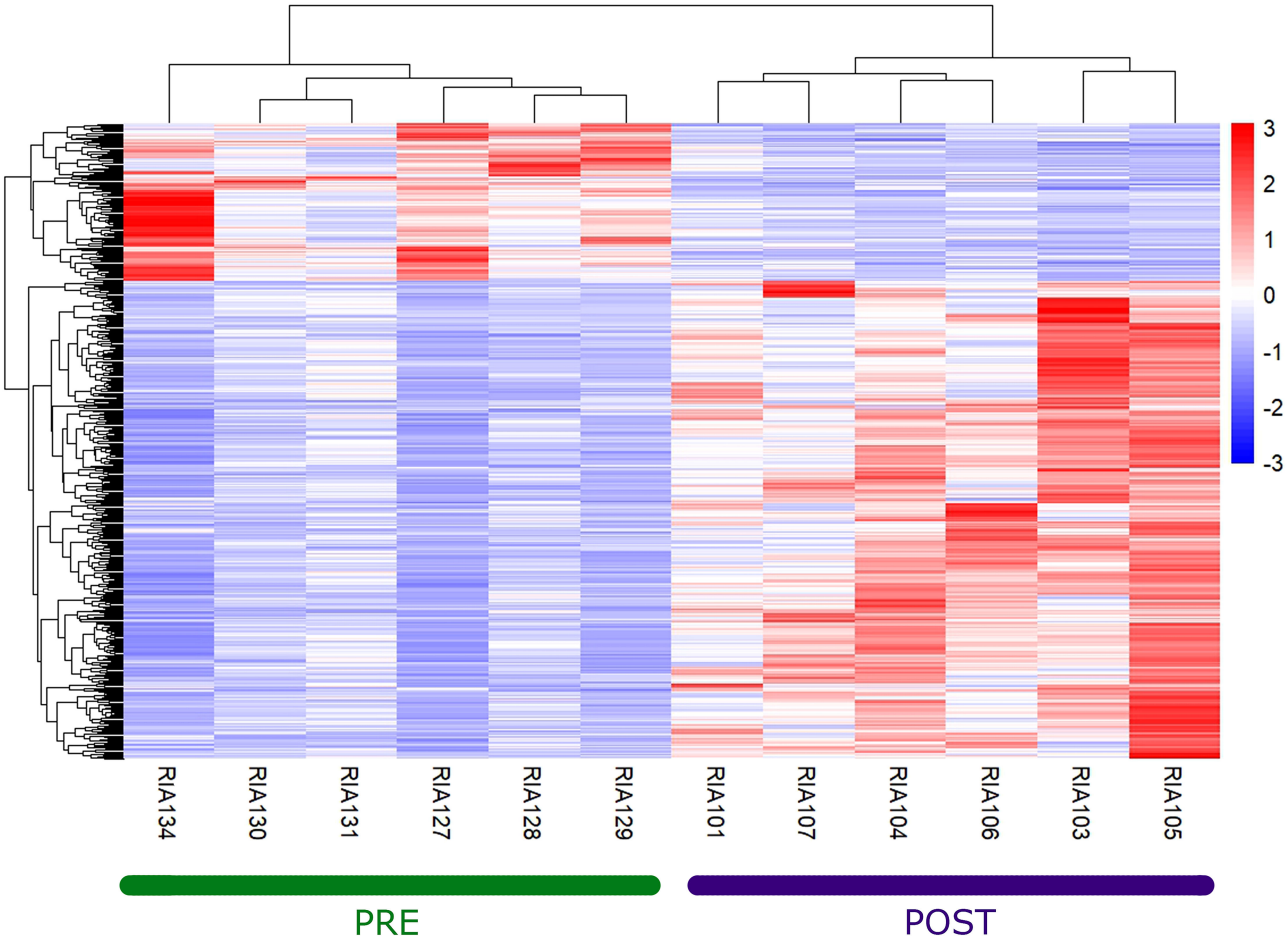
Heatmap of deregulated genes between pre- and post-therapy. The hierarchically clustered genes are represented by rows, and the samples are represented by columns. The red and blue colors indicate gene upregulation and downregulation, respectively.

Bulky RNA sequencing was performed in PBMCs of these patients to discriminate gene differentially expressed between before and after the administration of guselkumab (**Supplementary Figure 1**). Performing the quality check and then the filtering analyses (log2FoldChange > 1 and *p*_adj_ < 0.05) (**Supplementary Figure 2**) and filtering only coding mRNAs, we found 506 deregulated genes, of which 129 were upregulated and 377 were downregulated (**Figure 1**) between before and after the administration of guselkumab. In **Table 1**, the top 10 upregulated and downregulated genes are reported. We list the entire set of deregulated genes in **Supplementary Tables 1**, **2**.

**Table 1:** Top 10 upregulated and downregulated genes.

Depregulated genes	FoldChange	log2FoldChange	lfcSE	*p*-value	*p*_adj_
SYTL3	2.035251497	1.02520708	0.179317	1.08E−08	1.35E−05
CPT1A	2.066934732	1.047492833	0.223078	2.66E−06	0.000583
TMEM208	2.012805082	1.00920747	0.225197	7.41E−06	0.001086
KRT8P52	2.801802281	1.486355151	0.340035	1.24E−05	0.001481
GINS4	2.101707984	1.071562232	0.246373	1.37E−05	0.001579
TNFRSF13C	2.991907818	1.581065726	0.370181	1.95E−05	0.002014
BTBD6P1	3.551366023	1.82837406	0.432779	2.39E−05	0.002361
ZBTB32	4.381363037	2.13137976	0.509365	2.86E−05	0.002625
LAMA5	4.34668184	2.1199145	0.509216	3.14E−05	0.002808
POU5F1P5	2.679498168	1.42196283	0.342086	3.23E−05	0.002874
CCR2	−3.324075244	−1.732953039	0.260797	3.04E−11	5.78E−07
TPT1	−2.676707621	−1.420459561	0.230584	7.26E−10	2.77E−06
HSPE1P13	−9.323992966	−3.220947916	0.544015	3.21E−09	7.63E−06
MYCBP	−5.82854984	−2.543136981	0.429048	3.08E−09	7.63E−06
CMPK1	−4.061323098	−2.021949805	0.344425	4.34E−09	7.99E−06
CNIH1	−3.644073719	−1.865552145	0.317737	4.32E−09	7.99E−06
TMEM65	−2.295849806	−1.199028265	0.204595	4.61E−09	7.99E−06
ARL5A	−2.846598904	−1.509239226	0.263513	1.02E−08	1.35E−05
UBE2K	−3.212611531	−1.68374654	0.299001	1.79E−08	1.9E−05
GZMK	−4.807008048	−2.265139219	0.404158	2.09E−08	1.99E−05

**Figure 2:**
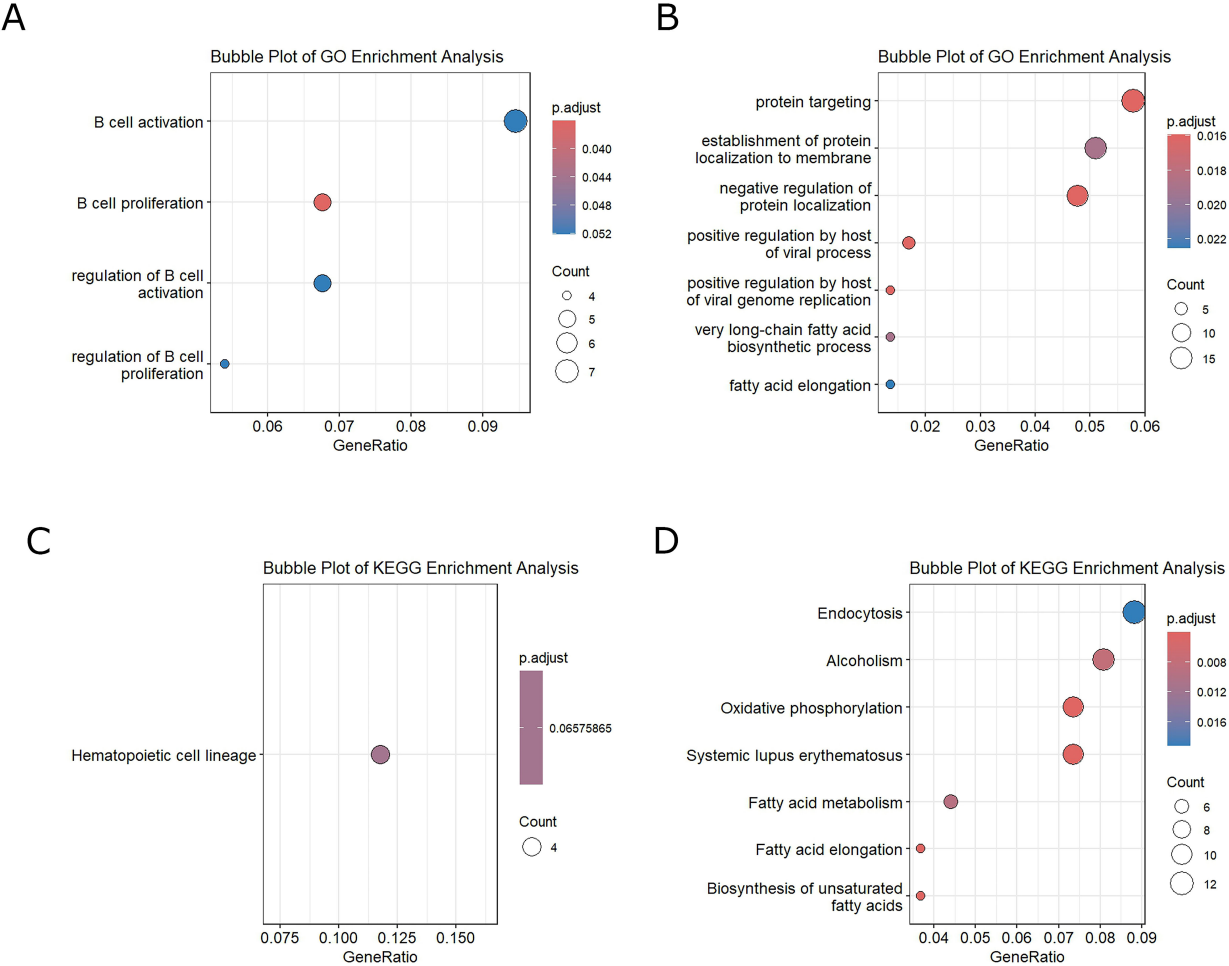
**(A)** GO enrichment-upregulated genes. **(B)** GO enrichment-downregulated genes. **(C)** KEGG enrichment-upregulated genes. **(D)** KEGG enrichment-downregulated genes.

In **Figure 2A**, we observe the enrichment analysis in the GO database of the upregulated processes, and in **Figure 2B**, we observe the downregulated ones. For KEGG enrichment, we performed the analysis for upregulated pathways (**Figure 2C**) and downregulated pathways (**Figure 2D**). In both enrichments, the significant upregulated processes were those related to B-lymphocyte activation (*p*_adj_ = 0.052) and proliferation (*p*_adj_ = 0.036). The downregulated processes in GO were Protein targeting (*p*_adj_ = 0.016) and Positive regulation by host of viral processes (*p*_adj_ = 0.018). The main processes downregulated in KEGG were endocytosis (*p*_adj_ = 0.020) and oxidative phosphorylation (*p*_adj_ = 0.004) (**Figure 2D**).

**Figure 3:**
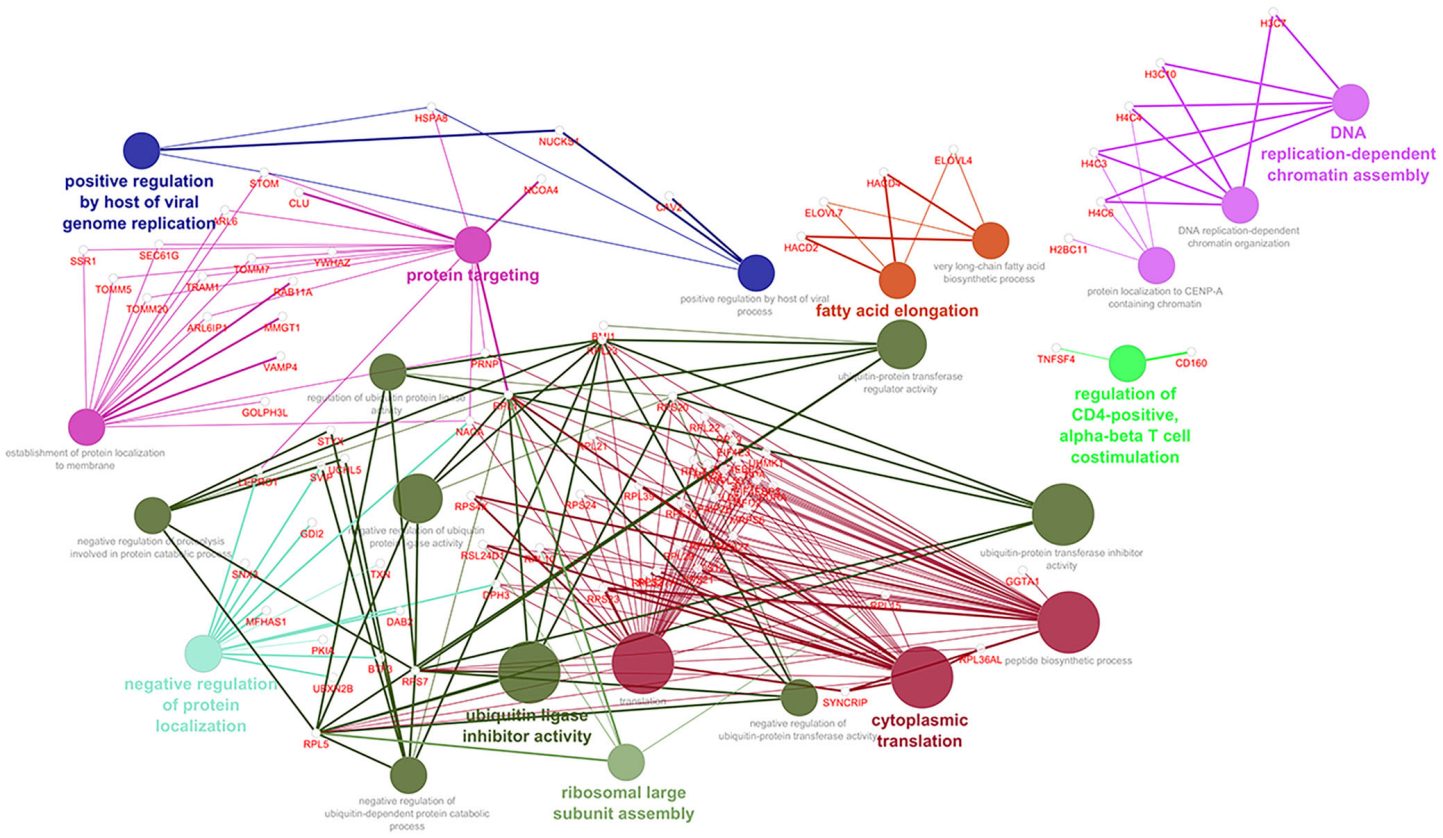
Enrichment of downregulated genes in GO biological processes.

Analyzing into the main processes querying GO Biological Processes, we identified the genes that contribute most to these deregulated pathways. In **Figure 3**, through Cytoscape, we consequently constructed an intricate network within the enriched pathway, and we reported the downregulated immune system network after the administration of guselkumab (**Supplementary Figures 3A, B**, respectively).

## Discussion

In this study, we provided a mechanistic scenario of the manipulated pathways following the administration of guselkumab in PSO and PSA by bulky RNA sequencing of PBMCs. This study may suggest how the inhibition of IL - 23 may have “pleiotropic” effects beyond the deregulated immune response underlying the development of PSO and PSA. In fact, the drug effects may be associated with some deeper consequences on cellular activities underpinning the clinical observed efficacy in treated patients.

The idea of the psoriatic disease continuum is increasingly prompted by multiple lines of evidence proposing a spectrum of inflammatory manifestations ranging from cutaneous to joint features according to a shared pathogenesis (4, 5, 8, 23). In this scenario, IL - 23 appears to play a key role by stimulating Th17 cells, and the inhibitors of this interleukin, including guselkumab, are effective in patients with PSO and PSA who do not respond to first-line therapies. However, the precise mechanisms of guselkumab efficacy are not fully understood yet (24). To elaborate further, RNA sequencing of PBMCs could offer a more detailed understanding of the molecular mechanisms involved, revealing how IL - 23 inhibition affects other pathological pathways.

In this study, we performed the assessment of transcriptome on PBMCs of six patients before and after treatment with guselkumab. We observed 558 deregulated mRNA, of which 135 were upregulated and 423 were downregulated post-therapy. In particular, we found an upregulation of SYTL3 gene recognized as increasing serum lipids and thus related to the risk of hyperlipidemia, which is one of the side effects in patients treated with guselkumab (25, 26). CPT1A was also found to be upregulated post-therapy. An interesting study in mice, investigating the action of rapamycin in autoimmune diseases and its role in inhibiting Th17 cells by increasing Treg cells, showed that CPT1A was upregulated following administration of the drug. Rapamycin was also able to abolish glycolysis in Th17 cells and to promote the fatty acid oxidation in Treg cells by upregulating the Treg/Th17 ratio (27). Furthermore, the TNFR13C gene, encoding for a TNF pathway receptor, showed increased expression levels after the inhibition of IL - 23. The role of this receptor in inflammatory diseases is still not entirely clear, although its interaction with other proteins is thought to play a crucial role in the presence of high amounts of autoantibodies (28).

The downregulated genes included CCR2, which is modulated in patients with PSA and is responsible for the Th2 immune response (29). Its downregulation after IL - 23 inhibition may suggest its ability to act not only on the IL23–Th17 axis but also on the Th2 response (30). Another interesting result is the downregulation of TPT1 after treatment with guselkumab. TPT1 is released in the serum of patients with PSO and is able to inhibit Treg cells, leading to an increase in type 1 macrophages. Its downregulation may suggest the role of IL - 23 inhibition in regulating the immune environment (31).

Our studies are in line with the work of Siebert et al., who observed an upregulation of B cells in a large case series. However, their work focuses on the enrichment of cell populations and not on the pathways that can modulate these populations and their functions (32).

Another important finding is the downregulation of signaling related to ethanol metabolism (named “alcoholism” in the KEGG database). Indeed, ethanol has been described in the literature as a factor that increases inflammatory cytokines and consequently increases the severity of PSO (33).

An interesting study by Liu et al. also conducted an analysis on PBMCs of healthy controls and patients with PSO and PSA. The single-cell analysis of this study showed that hemopoietic stem precursors were downregulated in patients with PSA together with genes encoding ribosomal proteins. In contrast, in patients with PSA, genes involved in metabolism and mitochondrial processes were upregulated (34). The results of our study may confirm that guselkumab could be also able to remodulate these deregulated processes in the disease context. In fact, after treatment, we noted an increase in processes regulating hemopoietic precursors and translational and transcriptional processes, and a decrease in pathways involved in metabolism, particularly those related to unsaturated fatty acids and oxidative phosphorylation that could re-establish a proper Treg/Th17 ratio. In particular, Th17 cells are strictly dependent on oxidative phosphorylation, and their downregulation has been shown in an *in vivo* study to attenuate their inflammatory response (35).

It is also interesting to note that fatty acids play an important role in PSA. In fact, patients with PSA have elevated serum levels of fatty acids that act as pro-inflammatory mediators, accentuating inflammation in the joints and psoriatic lesions, with the added risk of developing cardiovascular events and metabolic syndrome. The downregulation of unsaturated fatty acids after treatment therefore acts not only by reducing inflammation in the joints and psoriatic lesions but also by reducing the risk of comorbidities associated with this disease (36, 37). Another aspect that might be of interest is that guselkumab may be able to enhance the immune response to viral agents as we observed a downregulation of positive host regulation of viral processes (26). Thus, although, in the long term, the most frequently reported side effect after guselkumab therapy is infection, our findings may suggest that this drug could also be able to enhance the response against viruses, although further studies are needed to fully clarify this aspect. The increased likelihood of infection in these patients after guselkumab treatment is probably due to the fact that Th17 and IL17 play a crucial role in protecting against infection, and their inhibition leads to reduced protection against external agents (38, 39).

Despite providing further insights into the mechanism of action of guselkumab, our study has its share of limitations that affect the generalization of the results. The relatively low sample size, in spite of the evaluation of patients who were naïve to systemic immunomodulating therapies and diagnosed to have PSA with a duration < 2 years, may suggest the need of further studies to fully clarify this exploratory study. In addition, our bulky RNA sequencing has some limitations in evaluating the cell-specific heterogeneity despite its usefulness for understanding the average gene expression in a specific sample. A single-cell analysis to evaluate deregulated pathways in individual populations could elucidate the mechanisms of this drug and clarify the mechanisms that trigger side effects. A larger case series could also allow for an analysis of the differences between responders and non-responders. Taking together these observations, the hypothesis-generating nature of our study should be recognized in paving the way for further larger confirmatory-specific studies. In addition, keeping in mind the idea of the psoriatic disease continuum (1–5), further studies are needed to mechanistically compare the different clinical phenotypes in this context (i.e., patients with PSO but without PSA, patients with PSO and PSA, and patients with PSA without PSO) and how these diverse clinical presentations may benefit from the administration of the same drugs inhibiting IL - 23.

In conclusion, a deeper evaluation of manipulated mechanistic pathways by the inhibition of IL - 23 in patients with PSO and PSA was performed in our work. These hypothesis-generating results may provide the basis for further confirmatory studies to fully clarify the effects of IL - 23 in patients, underpinning the clinical efficacy of the administration of guselkumab in these patients. Our findings may suggest that behind the efficacy of IL - 23 inhibition in PSO and PSA, the deregulation of pathways beyond the immune system may be involved in a complex pathogenic scenario.

## Data Availability

The datasets presented in this study can be found in online repositories. The names of the repository/repositories and accession number(s) can be found in the article/**Supplementary Material**.

## Supplementary material

Supplementary Figure 1 PCA (Principal Component Analysis) Analysis in which by evaluating the set of all genes, it is possible to observe how the analyzed samples are distributed in space. With the Euclidean distances among all samples in each condition considered we performed PCA.

Supplementary Figure 2 Volcano Plot. In red are shown the significantly up-regulated genes with a Log2 Fold change ≥1, and in blue downregulated genes with a Log2 Fold change ≤1.

Supplementary Figure 3 Enrichment with GO ImmuneSystemProcesses **(A)** Upregulated Processes (in red Upregulated genes) **(B)** Downregulated Processes (in red Downregulated genes).

Supplementary Table 1 List of upregulated genes. The top 10 upregulated genes are marked in red.

Supplementary Table 2 List of downregulated genes. The top 10 downregulated genes are marked in red.

## References

[1] Griffiths CEM Armstrong AW Gudjonsson JE Barker JNWN. Psoriasis. Lancet. (2021) 397:1301–15. doi: 10.1016/S0140-6736(20)32549-6, PMID: 33812489

[2] FitzGerald O Ogdie A Chandran V Coates LC Kavanaugh A Tillett W. Psoriatic arthritis. Nat Rev Dis Primers. (2021) 7:59. doi: 10.1038/s41572-021-00293-y, PMID: 34385474

[3] Scarpa R. Psoriatic syndrome or psoriatic disease? J Rheumatol. (2020) 47:941. doi: 10.3899/jrheum.200051, PMID: 32358159

[4] Schett G Rahman P Ritchlin C McInnes IB Elewaut D Scher JU. Psoriatic arthritis from a mechanistic perspective. Nat Rev Rheumatol. (2022) 18:311–25. doi: 10.1038/s41584-022-00776-6, PMID: 35513599

[5] Sieminska I Pieniawska M Grzywa TM. The immunology of psoriasis-current concepts in pathogenesis. Clin Rev Allergy Immunol. (2024) 66:164–91. doi: 10.1007/s12016-024-08991-7, PMID: 38642273 PMC11193704

[6] Ruscitti P Esposito M Di Cola I Pellegrini C De Berardinis A Mastrangelo M. Cytokine profile characterization of naïve patients with psoriasis and psoriatic arthritis: implications for a pathogenic disease continuum. Front Immunol. (2023) 14:1229516. doi: 10.3389/fimmu.2023.1229516, PMID: 37520537 PMC10373502

[7] Ng BCK Lassere M. The role of the gastrointestinal microbiome on rheumatoid arthritis, psoriatic arthritis, ankylosing spondylitis and reactive arthritis: A systematic review. Semin Arthritis Rheumatol. (2025) 70:152574. doi: 10.1016/j.semarthrit.2024.152574, PMID: 39644691

[8] McGonagle D David P Macleod T Watad A. Predominant ligament-centric soft-tissue involvement differentiates axial psoriatic arthritis from ankylosing spondylitis. Nat Rev Rheumatol. (2023) 19:818–27. doi: 10.1038/s41584-023-01038-9, PMID: 37919337

[9] Nerviani A Boutet MA Tan WSG Goldmann K Purkayastha N Lajtos TA. IL-23 skin and joint profiling in psoriatic arthritis: novel perspectives in understanding clinical responses to IL-23 inhibitors. Ann Rheum Dis. (2021) 80:591–7. doi: 10.1136/annrheumdis-2020-218186, PMID: 33243781 PMC8053336

[10] Yang K Oak ASW Elewski BE. Use of IL-23 inhibitors for the treatment of plaque psoriasis and psoriatic arthritis: A comprehensive review. Am J Clin Dermatol. (2021) 22:173–92. doi: 10.1007/s40257-020-00578-0, PMID: 33301128 PMC7727454

[11] Fragoulis GE Siebert S. The role of IL-23 and the use of IL-23 inhibitors in psoriatic arthritis. Musculoskeletal Care. (2022) 20 Suppl 1:S12–21. doi: 10.1002/msc.1694, PMID: 36069174 PMC9825973

[12] Ghoreschi K Balato A Enerbäck C Sabat R. Therapeutics targeting the IL-23 and IL-17 pathway in psoriasis. Lancet. (2021) 397:754–66. doi: 10.1016/S0140-6736(21)00184-7, PMID: 33515492

[13] Gossec L Kerschbaumer A Ferreira RJO Aletaha D Baraliakos X Bertheussen H. EULAR recommendations for the management of psoriatic arthritis with pharmacological therapies: 2023 update. Ann Rheum Dis. (2024) 83:706–19. doi: 10.1136/ard-2024-225531, PMID: 38499325 PMC11103320

[14] Zhao S Fung-Leung WP Bittner A Ngo K Liu X. Comparison of RNA-Seq and microarray in transcriptome profiling of activated T cells. PloS One. (2014) 9:e78644. doi: 10.1371/journal.pone.0078644, PMID: 24454679 PMC3894192

[15] Kogenaru S Qing Y Guo Y Wang N. RNA-seq and microarray complement each other in transcriptome profiling. BMC Genomics. (2012) 13:629. doi: 10.1186/1471-2164-13-629, PMID: 23153100 PMC3534599

[16] Taylor W Gladman D Helliwell P Marchesoni A Mease P Mielants H. Classification criteria for psoriatic arthritis: development of new criteria from a large international study. Arthritis Rheumatol. (2006) 54:2665–73. doi: 10.1002/art.21972, PMID: 16871531

[17] Raychaudhuri SK Maverakis E Raychaudhuri SP. Diagnosis and classification of psoriasis. Autoimmun Rev. (2014) 13:490–5. doi: 10.1016/j.autrev.2014.01.008, PMID: 24434359

[18] Available online at: http://www.bioinformatics.babraham.ac.uk/projects/fastqc.

[19] Available online at: https://www.gencodegenes.org/human/ (Accessed September 13, 2024).

[20] Dobin A Davis CA Schlesinger F Drenkow J Zaleski C Jha S. STAR: ultrafast universal RNA-seq aligner. Bioinformatics. (2013) 29:15–21. doi: 10.1093/bioinformatics/bts635, PMID: 23104886 PMC3530905

[21] Love MI Huber W Anders S. Moderated estimation of fold change and dispersion for RNA-seq data with DESeq2. Genome Biol. (2014) 15:550. doi: 10.1186/s13059-014-0550-8, PMID: 25516281 PMC4302049

[22] Yu G Wang L-G Yan G-R He Q-Y. DOSE: an R/bioconductor package for disease ontology semantic and enrichmentanalysis. Bioinformatics. (2015) 31:608–9. doi: 10.1093/bioinformatics/btu684, PMID: 25677125

[23] Lobão B Lourenço D Giga A Mendes-Bastos P. From PsO to PsA: the role of TRM and Tregs in psoriatic disease, a systematic review of the literature. Front Med (Lausanne). (2024) 11:1346757. doi: 10.3389/fmed.2024.1346757, PMID: 38405187 PMC10884248

[24] Rocamora V Crespi L Ferran M Llamas-Velasco M Del Alcázar E Carrascosa JM. Guselkumab effectiveness and survival in patients with psoriasis and psoriatic arthritis: Multicenter analysis in daily clinical practice by the Spanish Psoriasis Group. Dermatol Ther. (2022) 35:e15865. doi: 10.1111/dth.15865, PMID: 36175141

[25] Zheng PF Yin RX Cao XL Guan YZ Deng GX Wei BL. *SYTL3*-*SLC22A3* single-nucleotide polymorphisms and gene-gene/environment interactions on the risk of hyperlipidemia. Front Genet. (2021) 12:679027. doi: 10.3389/fgene.2021.679027, PMID: 34367243 PMC8334725

[26] McInnes IB Rahman P Gottlieb AB Hsia EC Kollmeier AP Xu XL. Long-term efficacy and safety of guselkumab, a monoclonal antibody specific to the p19 subunit of interleukin-23, through two Years: results from a phase III, randomized, double-blind, placebo-controlled study conducted in biologic-naive patients with active psoriatic arthritis. Arthritis Rheumatol. (2022) 74:475–85. doi: 10.1002/art.42010, PMID: 34719872 PMC9305108

[27] Zhang J Jin H Xu Y Shan J. Rapamycin modulate treg/th17 balance via regulating metabolic pathways: A study in mice. Transplant Proc. (2019) 51:2136–40. doi: 10.1016/j.transproceed.2019.04.067, PMID: 31399190

[28] Roschke V Sosnovtseva S Ward CD Hong JS Smith R Albert V. BLyS and APRIL form biologically active heterotrimers that are expressed in patients with systemic immune-based rheumatic diseases. J Immunol. (2002) 169:4314–21. doi: 10.4049/jimmunol.169.8.4314, PMID: 12370363

[29] Caso F Saviano A Tasso M Raucci F Marigliano N Passavanti S. Analysis of rheumatoid- vs psoriatic arthritis synovial fluid reveals differential macrophage (CCR2) and T helper subsets (STAT3/4 and FOXP3) activation. Autoimmun Rev. (2022) 21:103207. doi: 10.1016/j.autrev.2022.103207, PMID: 36191778

[30] Bromley SK Larson RP Ziegler SF Luster AD. IL-23 induces atopic dermatitis-like inflammation instead of psoriasis-like inflammation in CCR2-deficient mice. PloS One. (2013) 8:e58196. doi: 10.1371/journal.pone.0058196, PMID: 23472158 PMC3589369

[31] Miao G Yang Y Yang X Chen D Liu L Lei X. The multifaceted potential of TPT1 as biomarker and therapeutic target. Heliyon. (2024) 10:e38819. doi: 10.1016/j.heliyon.2024.e38819, PMID: 39397949 PMC11471257

[32] Siebert S Sweet KM Ritchlin CT Hsia EC Kollmeier AP Xu XL. Guselkumab modulates differentially expressed genes in blood of patients with psoriatic arthritis: results from two phase 3, randomized, placebo-controlled trials. ACR Open Rheumatol. (2023) 5:490–8. doi: 10.1002/acr2.11589, PMID: 37553909 PMC10502816

[33] Farkas A Kemény L. Psoriasis and alcohol: is cutaneous ethanol one of the missing links? Br J Dermatol. (2010) 162:711–6. doi: 10.1111/j.1365-2133.2009.09595.x, PMID: 19922527

[34] Liu J Kumar S Hong J Huang ZM Paez D Castillo M. Combined single cell transcriptome and surface epitope profiling identifies potential biomarkers of psoriatic arthritis and facilitates diagnosis via machine learning. Front Immunol. (2022) 13:835760. doi: 10.3389/fimmu.2022.835760, PMID: 35309349 PMC8924042

[35] Franchi L Monteleone I Hao LY Spahr MA Zhao W Liu X. Inhibiting oxidative phosphorylation *in vivo* restrains th17 effector responses and ameliorates murine colitis. J Immunol. (2017) 198:2735–46. doi: 10.4049/jimmunol.1600810, PMID: 28242647 PMC5360504

[36] Looby N Roszkowska A Reyes-Garcés N Yu M Bączek T Kulasingam V. Serum metabolic fingerprinting of psoriasis and psoriatic arthritis patients using solid-phase microextraction-liquid chromatography-high-resolution mass spectrometry. Metabolomics. (2021) 17:59. doi: 10.1007/s11306-021-01805-3, PMID: 34137950 PMC8211611

[37] Peng L Chen L Wan J Liu W Lou S Shen Z. Single-cell transcriptomic landscape of immunometabolism reveals intervention candidates of ascorbate and aldarate metabolism, fatty-acid degradation and PUFA metabolism of T-cell subsets in healthy controls, psoriasis and psoriatic arthritis. Front Immunol. (2023) 14:1179877. doi: 10.3389/fimmu.2023.1179877, PMID: 37492568 PMC10363747

[38] Essigmann HT Hoffman KL Petrosino JF Jun G Aguilar D Hanis CL. The impact of the Th17:Treg axis on the IgA-Biome across the glycemic spectrum. PloS One. (2021) 16:e0258812. doi: 10.1371/journal.pone.0258812, PMID: 34669745 PMC8528330

[39] Matsuzaki G Umemura M. Interleukin-17 family cytokines in protective immunity against infections: role of hematopoietic cell-derived and non-hematopoietic cell-derived interleukin-17s. Microbiol Immunol. (2018) 62:1–13. doi: 10.1111/1348-0421.12560, PMID: 29205464

