# Supplementary figures and images for: Assessment of gene signatures following the inhibition of IL-23: a study to evaluate the mechanistic effects behind the clinical efficacy of guselkumab in patients with psoriatic arthritis
Source: Front Immunol. 2025 Sep 2;16:1672667. doi: 10.3389/fimmu.2025.1672667 (PMC12436422; doi:10.3389/fimmu.2025.1672667)

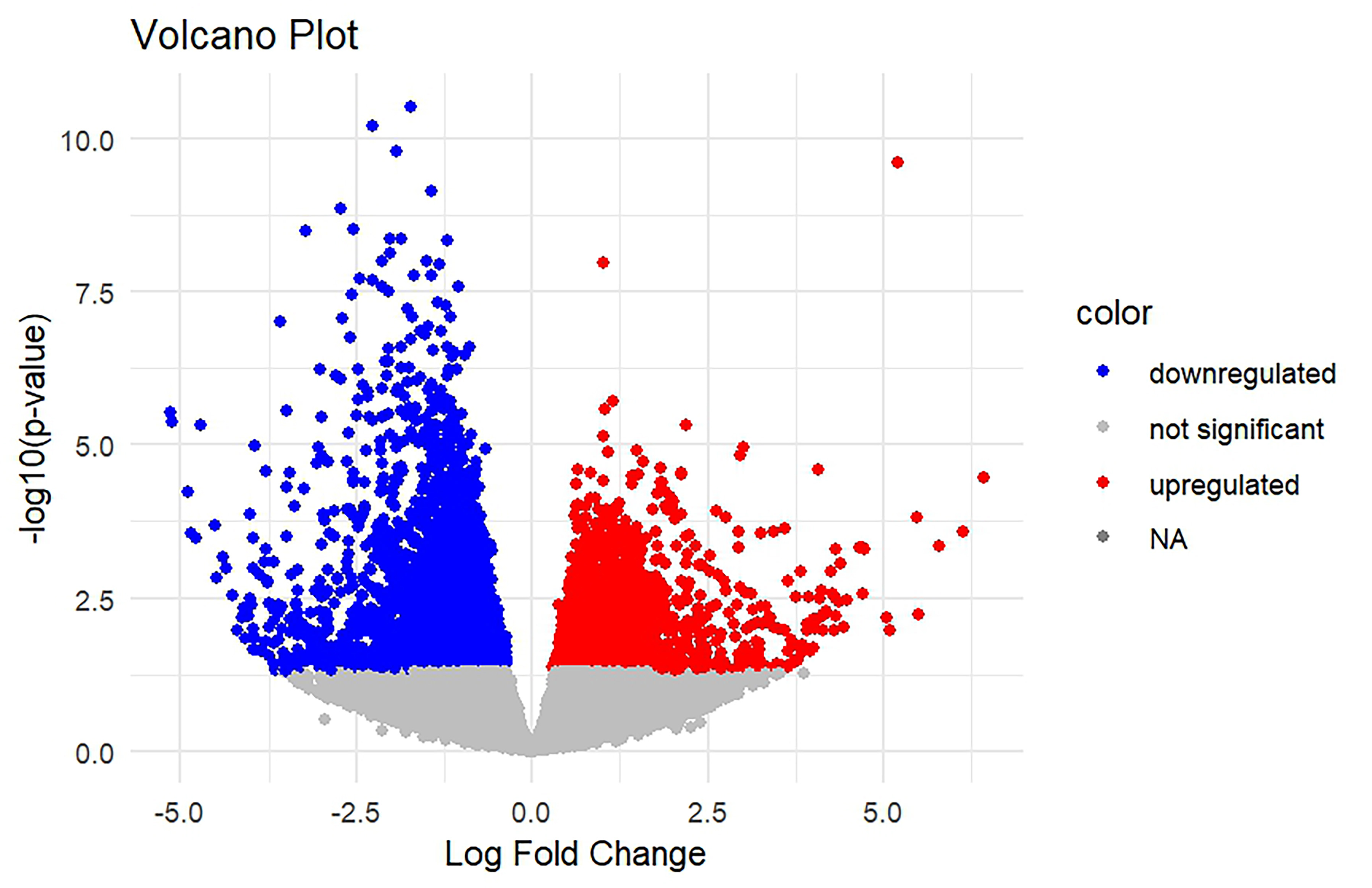

Supplement: Supplementary Figure 1 — PCA (Principal Component Analysis) Analysis in which by evaluating the set of all genes, it is possible to observe how the analyzed samples are distributed in space. With the Euclidean distances among all samples in each condition considered we performed PCA. [file DataSheet1.zip › Supplementary Figure 2.jpg]
